# 12-Deacetyl-12-epi-Scalaradial, a Scalarane Sesterterpenoid from a Marine Sponge Hippospongia sp., Induces HeLa Cells Apoptosis via MAPK/ERK Pathway and Modulates Nuclear Receptor Nur77

**DOI:** 10.3390/md18070375

**Published:** 2020-07-21

**Authors:** Mi Zhou, Bo-Rong Peng, Wenjing Tian, Jui-Hsin Su, Guanghui Wang, Ting Lin, Dequan Zeng, Jyh-Horng Sheu, Haifeng Chen

**Affiliations:** 1 Fujian Provincial Key Laboratory of Innovative Drug Target, School of Pharmaceutical Sciences, Xiamen University, Xiamen 361005, China.; zmxmuer@163.com (M.Z.); tianwj@xmu.edu.cn (W.T.); guanghui@xmu.edu.cn (G.W.); linting@xmu.edu.cn (T.L.); xmu-zdq@126.com (D.Z.); 2 National Museum of Marine Biology and Aquarium, Pingtung 944, Taiwan; pengpojung@gmail.com (B.-R.P.); x2219@nmmba.gov.tw (J.-H.S.); 3 Department of marine Biotechnology and Resources, National Sun Yat-sen University, Kaohsiung 804, Taiwan; 4 Graduate Institute of Marine Biology, National Dong Hwa University, Pingtung 944, Taiwan; 5 Department of Medical Research, China Medical University Hospital, China Medical University, Taichung 404, Taiwan; 6 Institute of Natural Products, Kaohsiung Medical University, Kaohsiung 807, Taiwan

**Keywords:** 12-deacetyl-12-*epi*-scalaradial, HeLa cells, apoptosis, Nur77, MAPK/ERK pathway

## Abstract

12-Deacetyl-12-*epi*-scalaradial, a scalarane sesterterpenoid from a marine sponge *Hippospongia sp*, has been reported to possess cytotoxic activity on HepG2, MCF-7, and HCT-116 cells. However, there is no research to indicate that 12-deacetyl-12-*epi*-scalaradial exhibited anticancer effect on cervical cancer HeLa cells. The aim of this study was to investigate the anticancer activity of 12-deacetyl-12-*epi*-scalaradial against HeLa cells and to explore the mechanism. The results from a methylthiazolyldiphenyl-tetrazolium (MTT) assay suggested that 12-deacetyl-12-*epi*-scalaradial suppressed the proliferation of HeLa cells and flow cytometry analysis showed 12-deacetyl-12-*epi*-scalaradial could induce the apoptosis of HeLa cells in dose- and time-dependent manner. Western blotting analysis demonstrated that 12-deacetyl-12-*epi*-scalaradial triggered apoptosis via mediating the extrinsic pathway and was found to suppress MAPK/ERK pathway which was associate with cancer cell death. Nur77, a critical number of orphan nuclear receptors, plays diverse roles in tumor development as a transcription factor and has been considered as a promising anticancer drug target. The dual-luciferase reporter assays suggested that 12-deacetyl-12-*epi*-scalaradial could selectively enhance the trans-activation activity of Nur77. Furthermore, Western blotting analysis and fluorescence quenching showed that 12-deacetyl-12-*epi*-scalaradial could induce the phosphorylation of Nur77 and interact with the ligand-binding domain (LBD) of Nur77. Our research confirmed 12-deacetyl-12-*epi*-scalaradial as a potential agent for cervical cancer therapy and provided a view that 12-deacetyl-12-*epi*-scalaradial may be a modulator of Nur77.

## 1. Introduction

Cancer, a hyper-proliferative disorder, ranks as the primary cause of mortality worldwide. Cervical cancer, with an increasing annual incidence and mortality rate, is one of the most frequently diagnosed cancers among women globally [1]. Even though the combination of chemotherapy and radiotherapy has had a certain effect on treating cervical cancer, it was accompanied with many side effects [2]. Therefore, it is worth persistently identifying small candidate molecules for the treatment of cervical cancer.

Nur77 (also named TR3, NR4A1, or NGFI-B), an orphan member of the nuclear receptor superfamily, participates in numerous cellular process such as growth, survival, and apoptosis. The biological effects of Nur77 are regulated by many factors, including its modulators [3,4]. Many reports showed that the natural modulators of Nur77 could lead to apoptosis in certain cancer cells. Cytosporone B, a polyketone from the marine fungi *Dothiorella sp.*, was the first natural agonist of Nur77and induced apoptosis via modulating the transactivation of Nur77 [5]. In addition, several other compounds targeting Nur77 derived from nature or synthesis were also found to interact with Nur77 and mediate the apoptosis of cancer cells [6,7,8,9,10]. Thus, Nur77 can be considered as a promising anticancer drug target. It is also of great significance to continually discover modulators of Nur77 from natural products which contribute to the development of Nur77-targeting anticancer drugs.

Marine natural products represent an extraordinarily rich source of chemical and biological diversity with unique chemical skeletons and outstanding functionalities [11,12]. A large number of terpenoids possessing novel carbon skeletons and a wide variety of biological activities were found in marine organisms, which was different from those present in terrestrial species. Scalarane sesterterpenoids, one of the unique types of terpenoid, are exclusively derived from sponges and shell less molluscs. Many of these compounds have been reported to show significant antitumor activity in vitro or in vivo [13,14,15,16,17,18], while most of their mechanisms of action remain unclear. Moreover, 12-deacetyl-12-*epi*-scalaradial, a scalarane sesterterpenoid, has been reported to show growth inhibitory activity against hepatocellular carcinoma, breast adenocarcinoma cells, and colorectal carcinoma [19]. However, whether 12-deacetyl-12-*epi*-scalaradial exhibited cytotoxicity against cervical cancer cells were still unreported so far. Therefore, in this work, we evaluated the antitumor effect and mechanism of 12-deacetyl-12-*epi*-scalaradial in HeLa cells, and further examined whether 12-deacetyl-12-*epi*-scalaradial modulated the transactivation activity and phosphorylation of Nur77. Further, we investigated the interaction between 12-deacetyl-12-*epi*-scalaradial and the ligand-binding domain (LBD) of Nur77.

## 2. Results

### 2.1. 12-Deacetyl-12-epi-scalaradial Inhibits HeLa Cell Proliferation

**Figure 1:**
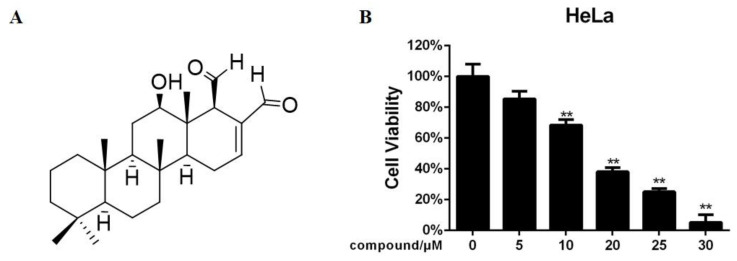
12-Deacetyl-12-*epi*-scalaradial inhibits HeLa cell proliferation. (**A**) The structure of 12-deacetyl-12-*epi*-scalaradial. (**B**) HeLa cells were treated with various concentration of 12-deacetyl-12-*epi*-scalaradial (0, 5, 10, 20, 25, 30 μM) for 48 h. After incubation, cell viability of HeLa cells was measured by MTT assay. The values are the mean ± SD for four independent replicates. (** *p* < 0.01, compared with untreated cells).

Briefly, 12-deacetyl-12-*epi*-scalaradial was isolated from marine sponge *Hippospongia sp* and its chemical structure was identified by a comparison with the literature [19] (Figure 1A). An MTT assay was used to investigate whether 12-deacetyl-12-*epi*-scalaradial showed cytotoxic effects against human cervical cancer cells. As shown Figure 1B, the HeLa cell viability was dose-dependently inhibited with an increasing concentration of 12-deacetyl-12-*epi*-scalaradial. Meanwhile, 12-deacetyl-12-*epi*-scalaradial exhibited significant inhibitory effects on HeLa cells under the concentration of 30 μM. Moreover, the IC_50_ value of 12-deacetyl-12-epi-scalaradial against HeLa cells was 13.74 μM, which is lower than the IC_50_ values of other cell lines in the reports with 36 μM for MCF-7, 23.4 μM for HepG2, and 27.1 μM for HCT-1116 [19]. The above data suggested that 12-deacetyl-12-*epi*-scalaradial possessed cytotoxic activities against HeLa cells, while HeLa cells seem to be more sensitive to 12-deacetyl-12-*epi*-scalaradial.

### 2.2. 12-Deacetyl-12-epi-scalaradial Induces HeLa Cells Apoptosis

**Figure 2:**
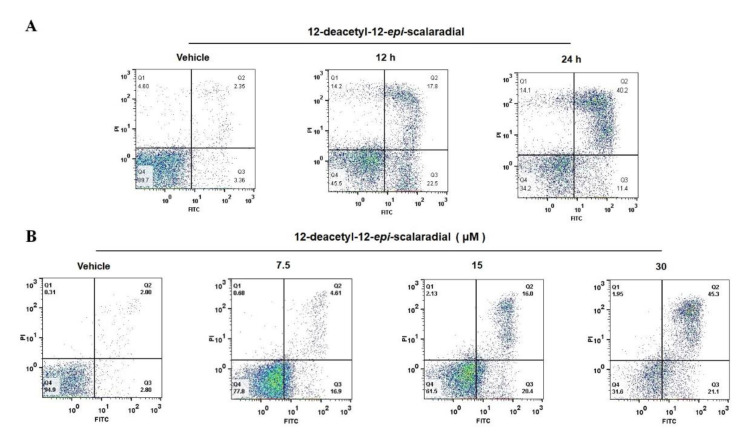
12-Deacetyl-12-*epi*-scalaradial induces HeLa cells apoptosis. (**A**) Flow Cytometry analysis via Annexin V/PI staining was used to investigate apoptosis induced by 12-deacetyl-12-*epi*-scalaradial. HeLa cells were treated with 30 μM compound for 12 or 24 h; (**B**) HeLa cells were treated with different concentrations of 12-deacetyl-12-*epi*-scalaradial (0, 7.5, 15, 30 μM) for 24 h.

Programmed cell death, also named apoptosis, is a critical process for erasing unneeded or unhealthy cells [20]. In many cancers, apoptosis was blocked by the abnormal activation of pro-apoptotic proteins or inhibition of anti-apoptotic pathways. Therefore, apoptosis induction is associated with the antiproliferation of tumour cells [21]. To investigate the ability of 12-deacetyl-12-*epi*-scalaradial to induce HeLa cells apoptosis, the cells were pretreated with compound (30 μM), and apoptosis was measured by flow cytometry. Indeed, 12-deacetyl-12-*epi*-scalaradial displayed a robust inducement of apoptotic activity in HeLa cells. Figure 2A shows the percentage of Annexin V-stained HeLa cells was 5.71% for the control, but the apoptosis percentages increased to 40.3% and 51.6% after treatment of compound (30 μM) for 12 and 24 h, respectively. Furthermore, the percentage of apoptotic cells was increased from 4.80% to 61.4% with a concentration of 12-deacetyl-12-*epi*-scalaradial in the range of 0 to 30 μM (Figure 2B). Thus, 12-deacetyl-12-*epi*-scalaradial significantly induced HeLa cells apoptosis in a time- and dose-dependent manner.

### 2.3. 12-Deacetyl-12-epi-scalaradial Induces PARP Cleavage and Activates Caspase Pathway in HeLa Cells

**Figure 3:**
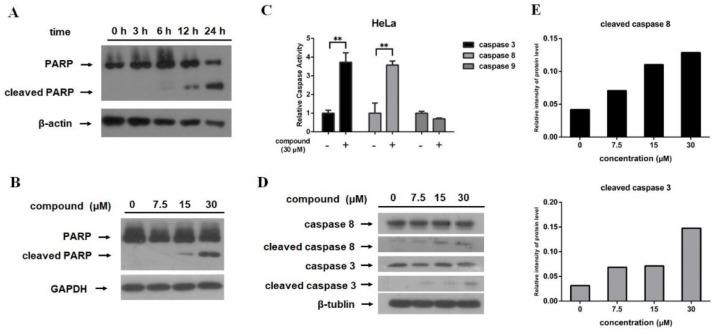
12-Deacetyl-12-*epi*-scalaradial induces PARP cleavage and activates caspase pathway in HeLa cells. (**A**) HeLa cells treated with 12-deacetyl-12-*epi*-scalaradial (30 μM) for 3, 6, 12, 24 h were analyzed by western blotting. (**B**) HeLa cells were incubated with different concentrations of 12-deacetyl-12-*epi*-scalaradial (0, 7.5, 15, 30 μM) for 24 h. The expression level of PARP and cleaved PARP were detected through western blotting (**C**) After treated with 12-deacetyl-12-*epi*-scalaradial (30 μM) for 12 h, caspases activities were measured by Caspase-Glo assay kit (Promega). The values are the mean + SD for three independent replicates. (**D**) Expression levels of caspase 3, caspase 8, cleaved caspase 3, cleaved caspase 8 and β-tublin were measured using western blotting after treated with 12-deacetyl-12-*epi*-scalaradial. (**E**) Quantification of western blotting in (**D**).

The presence of cleaved PARP is considered as one of the biomarkers for the detection of apoptosis in many cell lines [22,23,24]. In this study, Western blotting was used to identify PARP cleavage. As shown in Figure 3A, PARP cleavage was observed after HeLa cells were exposed to 30 μM compound for 6 h, which was more obvious when treated with compound for longer time. Dose response study showed that the PARP cleavage was stronger when treated with higher concentration of 12-deacetyl-12-*epi*-scalaradial for the same time (Figure 3B). These results were consistent with the flow cytometry analysis.

Caspases are crucial mediators of cell apoptosis. In mammals, while apoptosis occurs, caspases are activated in a protease cascade that leads to the activation or disablement of key structural proteins, signal pathways, and homeostatic and repair enzymes [25]. In order to investigate whether the caspases pathway contributed to the apoptotic effects of 12-deacetyl-12-*epi*-scalaradial, the Caspase-Glo assay kit was used to measure the caspases activity in HeLa cells with compound treatment. Figure 3C showed that the activities of caspase 3 and caspase 8 were increased after HeLa cells was exposed to 12-deacetyl-12-*epi*-scalaradial, while caspase 9 was not activated. As is known to all, activated caspase 8 is not only cleaved but also induce the cleavage of downstream effector caspase 3 [26]. Western blotting and relative intensity analysis further confirmed that the protein level of cleaved caspase 8 was increased dose dependently after treatment with 12-deacetyl-12-*epi*-scalaradial in HeLa cells, and cleaved caspase 3 was observed when HeLa cells were exposed to the compound in 30 μM (Figure 3D,E). These results were consistent with the analysis for cleaved PARP. The above data indicated that 12-deacetyl-12-*epi*-scalaradial induced PARP cleavage in a time- and concentration-dependent manner and it may induce apoptosis via the activation of caspase in human cervical cancer HeLa cells.

### 2.4. 12-Deacetyl-12-epi-scalaradial Suppresses MAPK/ERK Pathway

**Figure 4:**
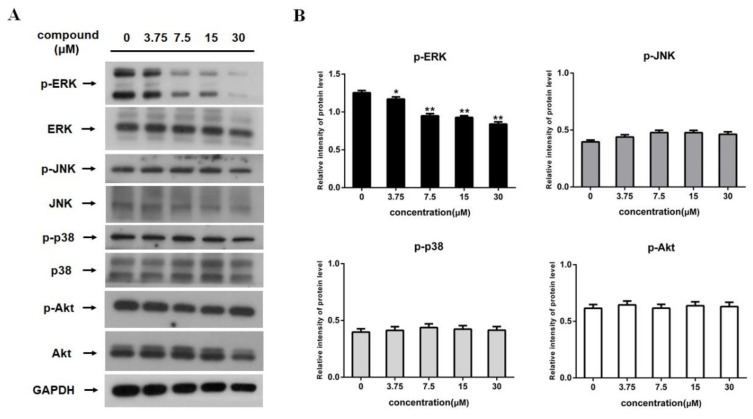
12-Deacetyl-12-*epi*-scalaradial suppresses MAPK/ERK pathway. (**A**) The expression levels of ERK, p-ERK, JNK, p-JNK, p38, p-p38, AKT, p-AKT and GAPDH were analyzed by western blotting. (**B**) Quantification of p-ERK, p-JNK, p-p38 and p-AKT in (A) (* *p* < 0.05, ** *p* < 0.01, compared with untreated cells).

The mitogen-activated protein kinase (MAPK) pathway plays crucial roles in various cellular functions, including growth, differentiation, and metastasis. MAPKs consists of three subfamilies including the extracellular signal-regulated kinase (ERK), c-Jun N-terminal kinase (JNK), and p38 kinase. The MAPK signaling pathway was closely involved in tumor cell apoptosis [27,28]. PI3K/Akt pathway is also associated with physiological process including survival and apoptosis in cancer cells [29]. In this work, the effects of 12-deacetyl-12-*epi*-scalaradial on proteins involved in the MAPK and PI3K/Akt pathway were investigated through western blotting assay. As shown in Figure 4A,B, the expression level of phosphorylation of ERK was reduced after treatment with 12-deacetyl-12-*epi*-scalaradial in a dose-dependent manner, while the expression level of p-JNK, p-p38, and p-Akt showed no difference. The above data demonstrated that 12-deacetyl-12-*epi*-scalaradial may suppress the MAPK/ERK pathway in HeLa cells. 

### 2.5. 12-Deacetyl-12-epi-scalaradial Modulates Trans-Activation Activity and Phosphorylation of Nur77, and Interacts with Nur77-LBD

**Figure 5:**
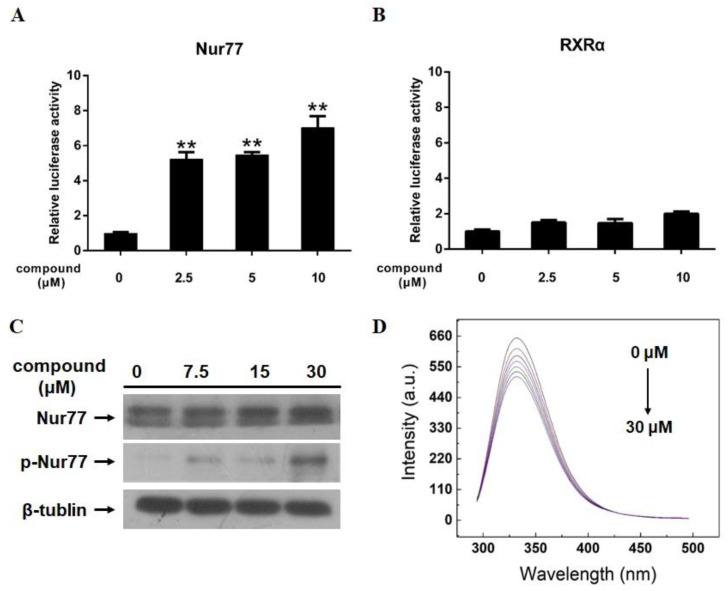
12-Deacetyl-12-*epi*-scalaradial stimulates trans-activation activity of Nur77 and interacts with Nur77-LBD. (**A**) After treated with different concentrations of 12-deacetyl-12-*epi*-scalaradial (0, 2.5, 5, 10 μM) for 12 h, the relative luciferase reporter-gene activity for Nur77 and RXRα were measured by dual-luciferase reporter gene assay. (**B**) After treated with different concentrations of 12-deacetyl-12-epi-scalaradial (0, 7.5, 15, 30 μM) for 12 h, the expression levels of Nur77, p-Nur77 and GAPDH were analysed by western blotting. (**C**) The expression levels of Nur77, p-Nur77 and β-tublin were analyzed by western blotting. (**D**) After treated with 12-deacetyl-12-*epi*-scalaradial (0–30 μM), the interacting affinity of compound toward Nur77-LBD was measured via fluorescence quenching technology at 298K.

Nur77, an important orphan member of the nuclear receptor superfamily, mediates survival or apoptosis in cancer cells. Some reports had shown that several signaling pathways, including the MAPK/ERK pathway, contributed to cell apoptosis through regulating the trans-activation function of Nur77 [30,31]. Meanwhile, a study showed that natural terpenoids exhibited antitumor activities via modulating the function of Nur77 [32]. Therefore, a dual-luciferase reporter gene assay, a method to identify small modulators of Nur77 [5,33,34,35], was used to evaluate the effect of 12-deacetyl-12-*epi*-scalaradial on trans-activation of Nur77. As shown in Figure 5A, the ascension of relative Nur77 luciferase reporter-gene activity was observed with increasing concentration of 12-deacetyl-12-*epi*-scalaradial. Our previous studies as well used dual-luciferase reporter gene assay to identify small modulators of Retinoid X Receptor-alpha (RXRα) [36,37,38,39], which is also an important nuclear receptor. Thus, to explore whether 12-deacetyl-12-*epi*-scalaradial selectively affected trans-activation activity of Nur77, the effect of compound on trans-activation activity of RXRα was measured. As a result, 12-deacetyl-12-*epi*-scalaradial has no influence on the trans-activation activity of RXRα with the same concentration (Figure 5B). Furthermore, the deactivation of MAPK/ERK pathway was reported to contribute to the phosphorylation of Nur77-mediated apoptosis [30,31]. Therefore, Western blotting was used to analyze the level of phosphorylation of Nur77 after treatment with 12-deacetyl-12-*epi*-scalaradial. Figure 5C exhibited that the expression level of phosphorylation of Nur77 increased clearly when exposed to high concentration of compound.

The phenyl alanine, tyrosine, and tryptophan in proteins are the donors of the fluorescence and the interaction between ligands and proteins will lead to the quenching of fluorescence. Thus, fluorescence quenching technology has been an effective way to study the binding of ligand to the target protein [40,41,42] and has been successfully used to investigate the interactions between natural modulators and the Nur77 [5,43]. In this study, the Nur77-LBD (ligand-binding domain) protein was purified and its interaction with 12-deacetyl-12-*epi*-scalaradial was measured by fluorescence quenching technology. As shown in Figure 5D, the fluorescence of Nur77-LBD protein was quenched in a dose-dependent manner after 12-deacetyl-12-*epi*-scalaradial treatment. The above results suggested that 12-deacetyl-12-*epi*-scalaradial could modulate trans-activation activity and phosphorylation of Nur77, and interact with Nur77-LBD.

## 3. Discussion

Cervical cancer is a growing worldwide health problem for women. There are several therapies for its treatment, such as surgery, radiotherapy and chemotherapy, but side effects remain a rigorous problem [1,2]. Therefore, it is urgent to develop effective drug for the treatment of cervical cancer. The marine natural products have shown extensive antitumor activity, and continuous attention has been drawn to discover drug candidates from marine organisms [44]. Scalarane sesterterpenoids, a unique type of terpenoids deriving from marine organisms, exhibit significant potential in the inhibition of cancer cells proliferation. Hyatelactam, a scalarane sesterterpenoid isolated from *Hyatella intestinalis*, showed inhibitory activity against human colon cancer HT-29 cells [16]. Another scalarane sesterterpenoid, hippospongide B, exhibited cytotoxicities against HCT-116, T-47D, K562, and DLD-1 tumor cells [17]. Heteronemin, also a scalarane sesterterpenoid from *Hyrtios sp.*, could induce the apoptosis of cancer cells via modulating the NF-κB and MAPKs signal pathways [4,45]. Even though scalarane sesterterpenoids are considered as a rich source of cancer therapeutic agents, their anticancer mechanism still needs to be investigated.

Briefly, 12-deacetyl-12-*epi*-scalaradial is a scalarane sesterterpenoid isolated from a marine sponge *Hippspongia sp*, which has been reported to inhibit the proliferation of several cancer cell lines containing HepG2, MCF-7 and HCT-116 [19]. However, the effects of 12-deacetyl-12-*epi*-scalaradial towards cervical cancer HeLa cells and its possible mechanism of antitumor activity remain largely unknown. Our results confirmed 12-deacetyl-12-*epi*-scalaradial inhibited the proliferation of HeLa cells (Figure 1B) with the IC_50_ value of 13.74 μM, which is lower than that of the other cell lines. Furthermore, we demonstrated that 12-deacetyl-12-*epi*-scalaradial could induce the apoptosis of HeLa cells (Figure 2 and Figure 3A,B). This further certificated that scalarane sesterterpenoids possessed broad antitumor activity, and showed promise as a leading cervical cancer therapeutic compound.

As is well known, the caspase pathway plays an essential role in cell apoptosis. Caspase enzymes family contains two major types of members, one type of apoptotic caspases is initiator caspases, which is activated through recruiting to signaling complexes as well as providing a link between cell signal pathway and apoptotic execution, such as caspase 8 and caspase 9. Caspase-8 is mediator of extrinsic apoptosis pathway, and the intrinsic apoptosis pathway leads to activation of caspase-9 [46]. Another type of caspases, named effector caspases, are activated by initiator caspases and most of the cellular substrates are cleaved by them. Among effector caspases, caspase-3 is the most frequently activated death protease in mammals, and poly (ADP-ribose) polymerase (PARP) is one of the downstream effectors of caspase 3 [47]. A CaspACE Assay System and Western blotting suggested that 12-deacetyl-12-*epi*-scalaradial simultaneously activated caspase 8 and caspase 3 enzymes (Figure 3C–E) to induce apoptosis of HeLa cells through extrinsic pathway. The MAPK and PI3K/Akt pathways are also important pathways associated with cancer cells apoptosis. The extracellular signal-regulated kinase (ERK), p38, and Jun N-terminal kinase (JNK) are three major subfamilies of the MAPK pathway. Among them, ERK and Akt signaling mediate the pro-survival effects of cancer cells, whereas the activation of JNK kinase and p38 kinase are involved in pro-apoptosis progress in cancer cells [48,49,50]. The data from this work showed that 12-deacetyl-12-*epi*-scalaradial induced the suppression of phosphorylation of ERK, contributed to the inhibitory effect on proliferation of HeLa cells (Figure 4A,B), and further confirmed that MAPK/ERK and caspase pathways played crucial roles in tumour cell apoptosis. Our result enriched the mechanism information concerning how scalarane sesterterpenoids exert antitumor effects.

The extrinsic pathway causes apoptosis through transmembrane receptor-mediated interactions including FasL and TRAIL. The interaction between FasL and TRAIL recruits cytoplasmic adapter proteins and caspase 8, resulting in the oligomerization and activation of caspase 8 that leads to apoptosis [51]. Several studies showed that FasL and TRAIL were promoted to enhance cancer cells apoptosis via Nur77 activated by DIM-C-pPhOCH_3_ compound [34,52], that indicated Nur77 could modulate extrinsic pathway to induce tumor cells apoptosis. Moreover, as a critical member of nuclear receptor superfamily, Nur77 is linked to various biological signaling pathways including MAPK and PI3K/Akt pathways in cancer cells. ERK pathway has been reported to depress Nur77-mediated apoptosis, and ERK inhibitor enhanced Nur77 to increase caspase 3 activity and induce apoptosis in HepG2 cells [31]. Our research indicated that 12-deacetyl-12-*epi*-scalaradial could selectively stimulate the trans-activation of Nur77 (Figure 5A,B). Furthermore, the phosphorylation of Nur77 is associated with MAPK/ERK pathway [30,31]. Our results showed that 12-deacetyl-12-*epi*-scalaradial suppressed the activation of MAPK/ERK pathway (Figure 4A,B) and induced the phosphorylation of Nur77 (Figure 5C). Interestingly, p38 and JNK pathways are also important signaling pathways for Nur77-modulated apoptosis. The phosphorylation of p38 and JNK enhance expression and translocation of Nur77, that lead to occurrence of cancer cells apoptosis [8,9,53]. The activation of PI3K/Akt pathway inhibits Nur77-induced apoptosis in cancer cells, respectively [54,55]. However, in this study, 12-deacetyl-12-*epi*-scalaradial was found to have no effects on JNK, p38, and Akt pathways. Previous reports and this work made us speculate that 12-deacetyl-12-*epi*-scalaradial may induce HeLa cell apoptosis by modulating Nur77 to mediate caspase and MAPK/ERK pathways. We also used fluorescence quenching technology to investigate interaction between 12-deacetyl-12-*epi*-scalaradial and Nur77-LBD (Figure 5D). However, regrettably, due to the insufficient amounts of compounds, we couldn’t further investigate the specificity of 12-deacetyl-12-*epi*-scalaradial binding to Nur77. These data showed the possibility of scalarane sesterterpenoids being a small modulator of Nur77 and provided a new direction for discovering natural modulators of Nur77. 

In conclusion, our results demonstrated that 12-deacetyl-12-*epi*-scalaradial, which is a scalarane sesterterpenoid, possessed anticancer activity against human cervical cancer HeLa cells via inducing apoptosis. Additionally, our data suggested that 12-deacetyl-12-*epi*-scalaradial may induce apoptosis through extrinsic pathway and MAPK/ERK pathway. Furthermore, 12-deacetyl-12-*epi*-scalaradial modulated the trans-activation activity and phosphorylation of Nur77 and interacted with Nur77-LBD. Our finding provides evidence about 12-deacetyl-12-*epi*-scalaradial as a potential agent of cervical cancer therapy and a small modulator of Nur77 for the first time.

## 4. Materials and Methods 

### 4.1. Chemicals and Reagents

HeLa cell line was purchased from the China Cell Bank of the Institute of Biochemistry and Cell Biology in Shanghai, China. Methylthiazolyldiphenyl-tetrazolium bromide (MTT) was purchased from Aladdin, China. Annexin V-FITC Apoptosis Detection Kits was purchased from Vazyme, China. Caspase-Glo assay kit was purchased from Promega, USA.

### 4.2. Isolation of Natural Products

Briefly, 12-deacetyl-12-*epi*-scalaradial was isolated from *Hippospongia sp.* The fresh sponge (1.2 kg wet weight) was extracted with EtOAc. The crude extract fraction (15.3 g) was separated on silica gel and eluted with using the mixtures of n-hexane/ EtOAc to obtain fractions 1-13. Purification from fraction 9 (5.4551 g) through repeated preparative TLC with n-Hexane–Acetone (10:1) afforded 12-deacetyl-12-*epi*-scalaradial. 

### 4.3. Cell Cultures

HeLa cells were cultured in Eagle’s Minimum Essential Medium (MEM) supplemented with 10% FBS in a humidified atmosphere containing 5% CO_2_ at 37 °C.

### 4.4. Cytotoxic Activity

Anti-proliferative activity of compound was tested by MTT assay. HeLa cells were seeded into 96-well-plate at a density of 5 × 10^3^ cells per well and allowed to settle 12 h. Then, the cells were treated with varied concentrations of 12-deacetyl-12-*epi*-scalaradial. After 48 h, 15 μL of MTT reagent and 60 μL MEM were added and incubated for 4 h. After removing the supernatant, the transformed crystals were dissolved in DMSO (100 μL) and measured at 490 nm using microplate reader (Thermo Multiskan MK3, Thermo Scientific, Helsinki, Finland). The cell proliferation-inhibition rate (%) was calculated as follows:

Growth Rate (%) = [(ODsample-ODblank)/(ODcontrol-ODblank)] × 100%

### 4.5. Flow Cytometry Analysis

The apoptotic rate of HeLa cells was assessed by flow cytometry using the Annexin-V/FITC Apoptosis Detection Kit according to manufacturer’s protocol. Cells were seeded into 6-well plates at a density of 1 × 10^5^ cells per well for 24 h. Cells were digested by trypsin with no EDTA and collected by centrifugation and resuspended in 100 μL of Binding buffer (1×) after 12-deacetyl-12-*epi*-scalaradial treatment. Finally, HeLa cells were stained with Annexin-V/FITC and PI for 10 min at room temperature in the dark. The stained cells were diluted with 1× binding buffer and analyzed by flow cytometry. Data were analyzed with FlowJo 10.

### 4.6. Western Blot Analysis

After treated with 12-deacetyl-12-*epi*-scalaradial, HeLa cells were lysed in RIPA buffer. Equal amounts of protein lysates were separated by SDS-PAGE and transferred onto polyvinylidene difluoride membranes. The membranes were blocked with 5% no-fat milk in TBST for 1 h, then incubated with primary antibodies at 4 °C overnight. After washing three times with TBST, the membranes were detected with secondary antibodies for 1 h under room temperature. The final immunoreactive products were detected by using an enhanced chemiluminescence system and examined by densitometric analysis.

### 4.7. Caspase Activation Activity

Caspase-3, -8, and -9 activities were measured using a Caspase-Glo assay kit (Promega) according to manufacturer’s protocol. HeLa cells were seeded in 96 well-culture dish and treated with 12-deacetyl-12-*epi*-scalaradial (30 μM). After 12 h, 100 µl Caspase-Glo Reagent were added to each well 12 h later. After 1 h incubation, the cells were measure the luminescence of each sample in a plate-reading luminometer as directed by the luminometer manufacturer.

### 4.8. Dual-Luciferase Reporter Assay

The 293T cells (293T) were cultured at 37 °C in DMEM with 10% FBS for 24 h and then seeded at a density of 1 × 10^4^ cells per well in 96-well plates. Three plasmids, namely pGL5-NURE luciferase reporter vector (15 ng/mL), myc-Nur77 vector (15 ng/mL), and pC DNA-Renila (15 ng/mL), were co-transfected by Liposome 2000. As to RXRα transcriptional activities, there are two plasmids, pGL5 luciferase reporter vector (30 ng/mL) and pGAL4-RXRα-LBD vector (15 ng/mL). After 12 hours, cells were incubated with the compounds at different concentrations for 12 h. The activities of firefly luciferase and renilla luciferase were measured using the Dual-Luciferase Assay System Kit (Promega). 

### 4.9. Nur77-LBD Protein Purified

The ligand-binding domain (LBD) of human Nur77 (genes 367–598) was cloned as N-terminal histidine-tagged fusion protein in pET15b vector and transformed to E. coli BL21(DE3). Cells were grown at 37 °C in LB medium until OD_600_ = 0.6–0.8 and then protein expression was induced by 1 mM IPTG. After sustained at 25 °C for 16 h, cells were harvested and sonicated. The target protein was purified using Ni^2+^-NTA agarose column at low temperatures. 

### 4.10. Fluorescence Quench Analysis

The fluorescence experiment was performed using a Cary Eclipse Fluorescence Spectrophotometer (Varian). Fluorescence spectra (300–500 nm) were conducted via excitation wavelength of 289 nm and slit widths to 10/10 nm at 289T.

### 4.11. Statistical Analysis

The values are presented as mean ± SD and were analyzed statistically with student’s t-tests for simple comparisons between groups, one-way analyses of variance (ANOVA) for more than two groups, and Tukey’s multiple comparisons test using GraphPad Prism 6.0. Differences were considered statistically significant at *p* < 0.05.
